# A Functional Ubiquitin-Proteasome System is Required for Efficient Replication of New World Mayaro and Una Alphaviruses

**DOI:** 10.3390/v11040370

**Published:** 2019-04-23

**Authors:** Yessica Y. Llamas-González, Dalkiria Campos, Juan M. Pascale, Juan Arbiza, José González-Santamaría

**Affiliations:** 1 Grupo de Biología Celular y Molecular de Arbovirus, Instituto Conmemorativo Gorgas de Estudios de la Salud, Panamá 0816-02593, Panama; qfb.y.llamas@gmail.com (Y.Y.L.-G.); dcampos@gorgas.gob.pa (D.C.); 2 Programa de Doctorado en Ciencias Biologicas, Universidad de la República, Montevideo 11200, Uruguay; 3 Dirección de Investigación y Desarrollo Tecnológico, Instituto Conmemorativo Gorgas de Estudios de la Salud, Panamá 0816-02593, Panama; jmpascale@gorgas.gob.pa; 4 Escuela de Medicina, Universidad de Panamá, Panamá, Panama; 5 Seccción de Virología, Facultad de Ciencias, Universidad de la República, Montevideo 11400, Uruguay; jarbiza@fcien.edu.uy; 6 Dirección de Investigación, Universidad Interamericana de Panamá, Panamá 9865, Panama

**Keywords:** alphaviruses, Mayaro, Una, ubiquitin-proteasome system, inhibition, replication, viral proteins

## Abstract

Mayaro (MAYV) and Una (UNAV) are emerging arboviruses belonging to the *Alphavirus* genus of the *Togaviridae* family. These viruses can produce febrile disease with symptoms such as fever, headache, myalgia, skin rash and incapacitating poly-arthralgia. Serological studies indicate that both viruses are circulating in different countries in Latin America. Viruses need the host cell machinery and resources to replicate effectively. One strategy to find new antivirals consists of identifying key cellular pathways or factors that are essential for virus replication. In this study, we analyzed the role of the ubiquitin-proteasome system (UPS) in MAYV and UNAV replication. Vero-E6 or HeLa cells were treated with the proteasome inhibitors MG132 or Lactacystin, and viral progeny production was quantified using a plaque assay method. In addition, the synthesis of viral proteins was analyzed by Western blot and confocal microscopy. Our results indicate that treatment with proteasome inhibitors decreases MAYV and UNAV protein synthesis, and also causes a significant dose-dependent decrease in MAYV and UNAV replication. Proteasome activity seems to be important at the early stages of MAYV replication. These findings suggest that the ubiquitin-proteasome system is a possible pharmacological target to inhibit these neglected alphaviruses.

## 1. Introduction

Arboviruses are viruses transmitted by arthropods, mainly mosquitoes [1]. These viruses have a global distribution and to date, more than 500 species grouped into different families have been described [2]. Although many of these viruses are nonpathogenic, some of these viruses can cause mild to severe illness in humans and animals. [2,3]. Some of these viruses belong to the *Alphavirus* genus (*Togaviridae* family), including Chikungunya virus (CHIKV), Eastern equine encephalitis virus (EEEV), Venezuelan equine encephalitis virus (VEEV) and Mayaro virus (MAYV) [2,4].

MAYV is an emerging New World *Alphavirus* that causes the disease known as Mayaro fever [5,6]. This virus was first isolated from serum samples from forest workers in Trinidad in 1954 [7]. MAYV is transmitted through the bites of mosquitoes belonging to the *Haemagogus* genus in the rainforests of Central and South American countries [5]. However, some evidence has revealed that the urban vector *Aedes aegypti* is capable of transmitting this virus [8,9]. MAYV has been associated with acute febrile illness with symptoms including fever, headache, eye pain, myalgia, diarrhea, skin rash and arthralgia [10,11]. Serological and virological surveys suggest that MAYV has been circulating in different countries, including Brazil, Peru, French Guiana, Venezuela, Colombia, Ecuador, Panama and Bolivia [12,13,14,15,16,17,18,19,20]. Recently, a MAYV infection in a Haitian child has increased concerns about the potential expansion of this virus, as has happened with other viruses such as Chikungunya and Zika [21,22].

MAYV has an 11.5 kb positive sense single-stranded RNA genome with two open reading frames that encode for both the non-structural proteins (nsP1, nsP2, nsP3 and nsP4), which are implicated in transcription and replication of viral RNA, and the structural proteins (C, E3, E2, 6k and E1), some of which form part of the viral particle [6,23]. Knowledge about the MAYV pathogenesis is limited and currently there are no approved vaccines or antiviral molecules to combat this infection.

Una virus (UNAV) is another New World *Alphavirus* closely related to MAYV [24]. It has been isolated from different species of *Psorophora* mosquitos in Brazil, Panama, Surinam and Venezuela [19,25,26,27,28]. Several serological studies have demonstrated the circulation of UNAV in both non-human primates and human populations in some regions of Argentina and Paraguay [29,30,31]. However, the pathogenesis and symptoms of this virus are unknown.

Viruses are intracellular-obligate pathogens that have developed different strategies to replicate in host cells, including hijacking the ubiquitin-proteasome pathway (UPS) [32,33]. Ubiquitination consists of the conjugation of ubiquitin molecules to a lysine residue on a substrate protein [34]. The UPS regulates different cellular processes, such as protein degradation, DNA repair, receptor trafficking, immunity, cell cycle progression, autophagy and viral infection [35,36]. Not surprisingly, different viruses such as rotavirus (RotV), hepatitis E virus (HEV), African swine fever virus (ASFV), human cytomegalovirus (HCMV), porcine circovirus type 2 (PCV2), influenza A virus (IAV) and human respiratory syncytial virus (HRSV), exploit the UPS to favor their replication [37,38,39,40,41,42]. Moreover, several lines of evidence indicate that Alphaviruses manipulate the UPS to promote their replication. For example, nsP2 proteins from Old World Alphaviruses Sindbis virus (SINV), Selimki Forest virus (SFV) and CHIKV use a proteasome-dependent mechanism to provoke the degradation of Rpb1, a catalytic subunit of RNA polymerase II, which induces inhibition of cellular transcription [43]. Additionally, it has been reported that the capsid protein of VEEV is ubiquitinated, and this modification is important for its replication [44]. However, the role of the UPS in MAYV and UNAV replication has not yet been explored. The aim of this study was to evaluate the role of the UPS in MAYV and UNAV replication.

## 2. Materials and Methods

### 2.1. Cell culture, Virus Strains Propagation and Reagents

Vero-E6 (ATCC, CRL-1586) and HeLa cells (kindly provided by Dr. Carmen Rivas, CIMUS, Spain) were grown in Minimal Essential Medium (MEM) and supplemented with 2 mM of l-Glutamine, penicillin-streptomycin antibiotic solution and 10% fetal bovine serum (FBS; Gibco, Waltham, MA, USA). MAYV (AVR0565, San Martín, Perú) [24] and UNAV (BT-1495-3, Bocas del Toro, Panama) [26] strains were kindly provided by Dr. Robert Tesh from The World Reference Center for Emerging Viruses and Arboviruses (WRCEVA) at University of Texas Medical Branch (UTMB), USA. The viruses were propagated in Vero-E6 cells in MEM supplemented with 2% FBS. Supernatants of infected cells were collected, clarified and concentrated using Amicon Ultra centrifugal filters (Merck, Kenilworth, NJ, USA), and then titrated, aliquoted and stored at −80 °C until use. The proteasome inhibitors MG132 (Sigma-Aldrich, Saint Louis, MI, USA) and Lactacystin (Tocris, Minneapolis, MN, USA) were dissolved in Dimethyl sulfoxide (DMSO) and water, respectively, at a concentration of 10 mM. The stock solutions were aliquoted and stored at −20 °C. MG132 is a potent reversible inhibitor of the chymotryptic-like activity of the proteasome [45], and Lactacystin is an irreversible inhibitor that binds to the catalytic β subunits of the 20S proteasome [46]. Working solutions of the proteasome inhibitors were prepared in MEM at the indicated concentrations.

### 2.2. Cell Viability Assay

The toxicity of the proteasome inhibitors was evaluated using the 3-(4,5-Dimethyl-2-thiazolyl)-2,5-diphenyltetrazolium bromide (MTT) assay (Sigma-Aldrich, Saint Louis, MI, USA), following the manufacturer’s instructions. Briefly, 2.5 × 10^4^ Vero-E6 or HeLa cells were seeded in 96-well plates in MEM without phenol red. Then, the cells were treated with MG132 (0, 0.1, 1 and 10 μM) or Lactacystin (0, 0.5, 5 and 25 μM). At 24 or 48 h after treatment, 5 mg/mL of MTT solution in PBS was added to the cells and incubated for an additional 4 h. Formazan crystals were dissolved in a solution of 4 mM HCL and 10% Triton X-100 in isopropanol, and absorbance was measured at 570 nm using a microplate reader spectrophotometer (BioTek, Winooski, VT, USA). Results are expressed as the percentage of viable cells relative to the untreated control cells.

### 2.3. Plaque Assay

Virus yields in virus stock or supernatants of infected cells were quantified by plaque assay. Briefly, confluent monolayers of Vero-E6 cells in 6-well plates were infected with 10-fold dilutions of virus samples. One hour after incubation, the inoculum was removed, and an Agar-medium solution supplemented with 2% FBS was added. Infected cells were then incubated for 3 days at 37 °C under a 5% CO_2_ atmosphere. Finally, the agar was removed, and the cells were fixed with 4% formaldehyde solution in PBS and stained with 2% crystal violet dissolved in 30% methanol. The number of plaques were counted and expressed as plaque-forming units per milliliter (PFU/mL).

### 2.4. Immunofluorescence and Confocal Microscopy Analysis

HeLa cells seeded onto glass coverslips were infected with MAYV at a multiplicity of infection (MOI) of 1. At 24 h after infection, cells were fixed in 2% paraformaldehyde for 20 min, and permeabilized with 0.25% Triton-X100. Cells were then incubated in 2% bovine serum albumin solution in PBS for 20 min and stained overnight at 4 °C with a mouse immune ascitic fluid anti-MAYV antibody (kindly provided by Dr. Scott Weaver, WRCEVA, UTMB, USA). Finally, cells were washed with PBS buffer and incubated with a Goat anti-mouse secondary antibody, Alexa-Flour 488 conjugate (Invitrogen, Carlsbad, CA, USA), for one hour in the dark. Coverslips were mounted on slides with Prolong Diamond Antifade Mountant with Dapi (Invitrogen, Carlsbad, CA, USA), and the images were obtained with a FV1000 Flowview Confocal microscope (Olympus, Lombard, IL, USA). The pictures were exported and analyzed with ImageJ software [47]. The number of MAYV-positive cells were counted in at least 10 fields and represented as the percentage of positive cells.

### 2.5. Protein Analysis

Vero-E6 or HeLa cells were treated with proteasome inhibitors and infected with MAYV or UNAV. Untreated and uninfected cells served as controls. Confluent monolayers of the cells were lysed with Laemmli loading buffer with 10% β-mercaptoethanol (Bio-Rad, Hercules, CA, USA). Proteins were separated by SDS-PAGE, transferred to nitrocellulose membranes and incubated in a 5% non-fat milk solution in T-TBS buffer, as was previously reported [47]. Membranes were incubated overnight at 4 °C with the following primary antibodies: rabbit polyclonal anti-MAYV E1, rabbit polyclonal anti-MAYV nsP1 (both antibodies custom-made by GeneScript, Piscataway, NJ, USA), rabbit monoclonal anti-ubiquitin Lys-48 specific (clone Apu2, Cat. # 05-1307, Merck Millipore, Kenilworth, NJ, USA), rabbit monoclonal anti-GAPDH (D16H11, Cat. # 5174, Cell Signaling Technology, Danvers, MA, USA), and rabbit monoclonal anti-β-actin (D6A8, Cat. # 8457, Cell Signaling Technology, Danvers, MA, USA). The membranes were then washed three times in T-TBS buffer and incubated with HRP-conjugated goat anti-rabbit antibody (Cat. # 926-80011, LI-COR, Lincoln, NE, USA) at room temperature for 1 h. Finally, the membranes were incubated for 5 min in WesternSure ECL substrate (LI-COR, Lincoln, NE, USA), and the chemiluminescent signal was detected with a chemiluminescent scanner device (LI-COR, Lincoln, NE, USA).

### 2.6. Design, Generation and Validation of Polyclonal Antibodies Against MAYV E1 and nsP1 Protein

Synthetic DNA constructs encoding the first 400 amino acids of E1 protein (Q8QZ72, UniProt) or the full version of nsP1 protein (Q8QZ73, UniProt) of MAYV (Brazil strain), were expressed in bacteria and the purified recombinant proteins were used to immunize rabbits using a conventional protocol (GenScript, Piscataway, NJ, USA). Antibodies were validated by Western blot analysis using 50 ng of recombinant E1 or nsP1 proteins as positive controls. In addition, Vero cells were infected with MAYV, UNAV or CHIKV at an MOI of 1, and then protein extracts were analyzed by Western blot using pre-immune sera or purified antibodies.

### 2.7. Statistical Analysis

Experimental data of control and treated groups were analyzed with the Mann & Whitney test, *t*-Student test, One-way ANOVA and Two-way ANOVA using GraphPad Prism 7 software for Mac (La Jolla, CA, USA). All experiments were performed at least 3 times with 3 or 4 replicates. A *p* value < 0.05 was considered statistically significant.

## 3. Results

### 3.1. Effect of Proteasome Inhibitors in Vero-E6 and HeLa Cells

**Figure 1:**
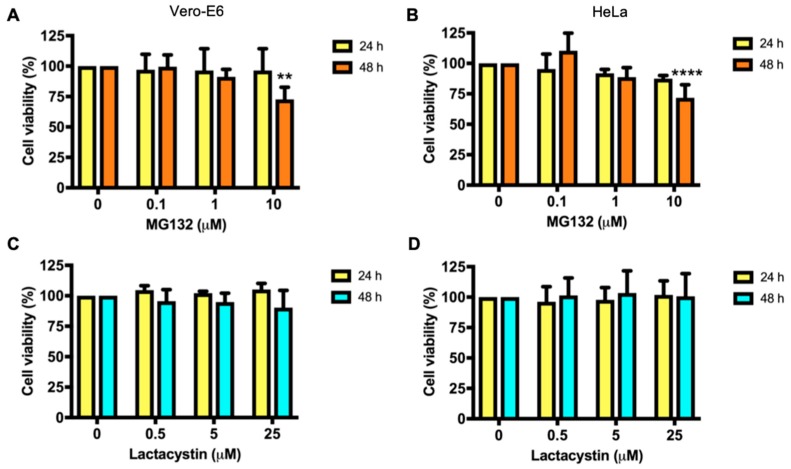
Cytotoxicity of proteasome inhibitors in Vero-E6 and HeLa cells. Vero-E6 (**A**,**C**) or HeLa (**B**,**D**) cells were treated with the indicated concentrations of MG132 or Lactacystin. In control cells, DMSO (0.1%) or water was added. After 24 or 48 h of treatment, cell viability was analyzed using an MTT assay. Cell viability data were analyzed with the Two-way ANOVA test using GraphPad Software and are shown as mean ± SD. Statistically significant differences are indicated: ** *p* < 0.01, **** *p* < 0.0001.

In order to evaluate the putative cytotoxicity of the proteasome inhibitors, we treated Vero-E6 and HeLa cells with the indicated concentrations of both MG132 and Lactacystin. At 24 or 48 h after treatment, we analyzed cell viability using an MTT assay. We observed nearly a 25% reduction in Vero-E6 and HeLa cell viability at 48 h after treatment with the highest dose of MG132 (10 μM) (Figure 1A,B). In contrast, we did not observe any cytotoxic effect of Lactacystin on either Vero-E6 or HeLa cells, regardless of drug concentration or incubation time (Figure 1C,D). Therefore, treatment with the proteasome inhibitors was carried out for 24 h in the subsequent experiments.

To check the activity of the proteasome inhibitors, we also analyzed the presence of polyubiquitinated proteins in treated Vero-E6 or HeLa cells using a Western blot with an antibody that only recognizes the polyubiquitin chains linked through the 48th lysine residue (K48) of ubiquitin [48]. As expected, treatment with the proteasome inhibitors induced a dose-dependent accumulation of K48-linked polyubiquitinated protein in both Vero-E6 (Figure S1A,C) and HeLa cells (Figure S1B,D).

### 3.2. Treatment with Proteasome Inhibitors MG132 or Lactacystin Reduces MAYV Progeny Production in Vero-E6 and HeLa Cells

**Figure 2:**
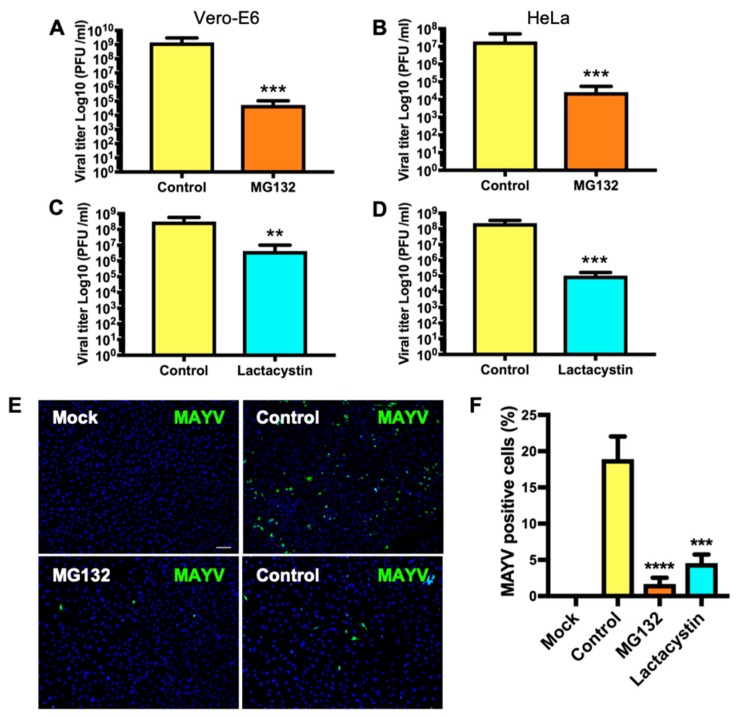
Proteasome inhibition reduces MAYV progeny production. Vero-E6 (**A**,**C**) or HeLa (**B**,**D**) cells were pre-treated with 10 μM of MG132 or 25 μM of Lactacystin. At 1 h after treatment, the media was removed and the cells were infected with MAYV at an MOI of 1 (Vero-E6) or 10 (HeLa). After 1 h of virus absorption, fresh media supplemented with the inhibitors was added to the cells. In control cells, DMSO (0.1%) or water was added. The production of progeny virus in the supernatants after 24 h of infection was quantified by plaque assay. Viral titers were expressed as plaque-forming units per milliliter (PFU/mL). (**E**) HeLa cells grown onto glass coverslips were treated or untreated with the proteasome inhibitors and infected with MAYV at an MOI of 1. After 24 h of infection, the cells were fixed with 2% paraformaldehyde and stained with a mouse anti-MAYV antibody, followed by an Alexa-Flour 488 anti-mouse secondary antibody. The images were obtained with a confocal microscope and analyzed using ImageJ software. Scale bar, 100 µm. (**F**) The percentage of MAYV-positive cells following infection of proteasome inhibitor-treated and untreated cells are shown. Statistically significant differences are indicated: ** *p* < 0.01; *** *p* < 0.001; **** *p* < 0.0001.

To test the effect of the proteasome inhibitors on MAYV replication, we pre-treated Vero-E6 or HeLa cells with the indicated concentrations of MG132 or Lactacystin. At 1 h after treatment, cells were infected with MAYV at an MOI of 1 or 10. After 1 h of virus absorption, we removed the inoculum and added fresh medium supplemented with the inhibitors. At 24 h after infection, we collected the supernatants and measured the progeny viruses produced by the infected cells. We observed a significant reduction in viral production with MG132 treatment in both cell lines (Figure 2A,B). Similar results were observed after Lactacystin treatment (Figure 2C,D). To verify these results, we carried out immunofluorescence staining of MAYV-infected HeLa cells that were treated or untreated with the proteasome inhibitors using anti-MAYV antibody. We observed a significant reduction in the percentage of MAYV-positive cells after treatment with the proteasome inhibitors (Figure 2E,F). Together, these data indicate that proteasome inhibition reduces the MAYV progeny yield.

### 3.3. Decrease in MAYV Titers in Response to Proteasome Inhibitors Occurs in a Dose-Dependent Manner

**Figure 3:**
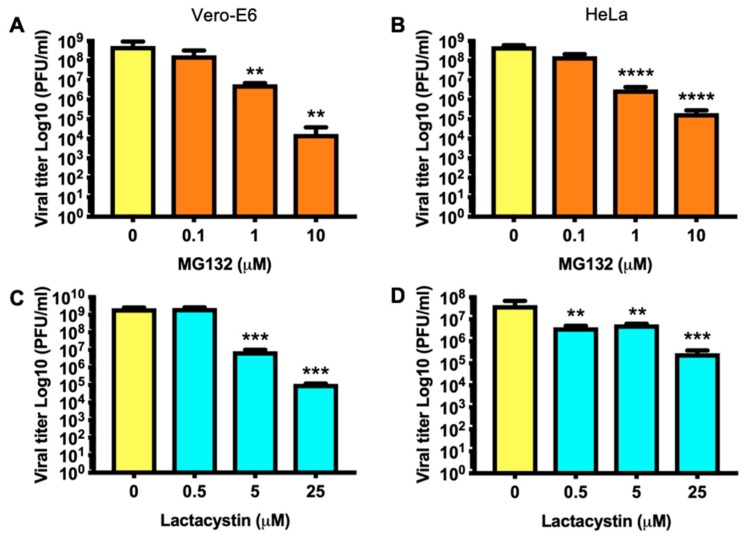
Proteasome inhibitors reduce MAYV progeny production in a concentration-dependent manner. Vero-E6 (**A**,**C**) or HeLa (**B**,**D**) cells were pre-treated with the indicated concentrations of proteasome inhibitors. In control cells, DMSO (0.1%) or water was added. At 1 h after treatment, cells were infected with MAYV as described above. Titration of the progeny virus in cell supernatants was carried out by plaque assay. Viral titers were expressed as plaque-forming units per milliliter (PFU/mL). Data were analyzed with the One-way ANOVA test using GraphPad software and are shown as mean ± SD. Statistically significant differences are indicated: ** *p* < 0.01; *** *p* < 0.001; **** *p* < 0.0001.

To further characterize the effect of the proteasome inhibitors on MAYV replication, we quantified virus production in supernatants of Vero-E6 or HeLa cells treated with increasing concentrations of MG132 or Lactacystin. As shown in Figure 3, there was a significant dose-dependent reduction in viral titers in both Vero-E6 (panels A and C) and HeLa (panels B and D) cells.

### 3.4. Proteasome Activity is Necessary in the First Stage of the MAYV Replication Cycle

**Figure 4:**
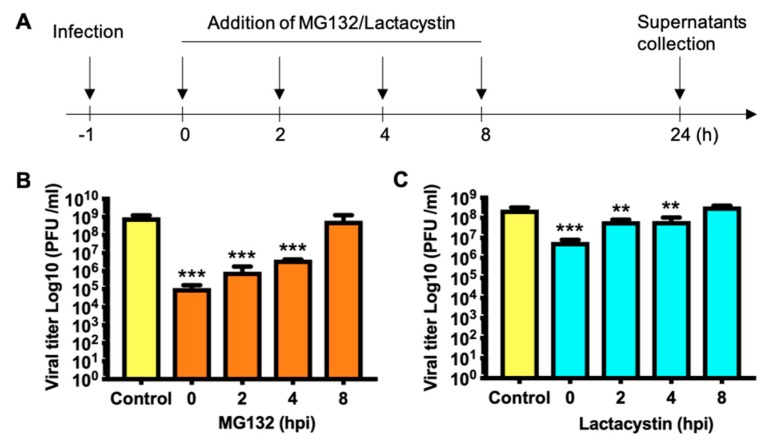
MAYV requires proteasome activity at the early stages of infection. (**A**) Schematic representation of the experiment applying the proteasome inhibitors at different times after infection. HeLa cells were infected with MAYV as described above. MG132 (**B**) or Lactacystin (**C**) was added at the indicated time after infection. In control cells, DMSO (0.1%) or water was added at 0 h. At 24 h of infection we assessed viral yield production in the cell supernatants. Viral titers were expressed as PFU/mL. Data were analyzed with the One-way ANOVA test using GraphPad software and are shown as mean ± SD. Statistically significant differences are indicated: ** *p* < 0.01, *** *p* < 0.001.

To determine which steps in the virus life cycle are targeted by proteasome inhibitors, we evaluated MAYV production after applying the inhibitors at different time points after infection (Figure 4A). Our results reveal that MG132 treatment only promoted a significant decrease in viral progeny production when the proteasome inhibitor was added during the first 4 h of infection (Figure 4B). Furthermore, in Lactacystin-treated cells, we observed similar results (Figure 4C). These results suggest that MAYV requires proteasome activity at the early stages of infection in order to promote viral replication.

### 3.5. Proteasome Inhibition Impairs MAYV Viral Protein Synthesis

**Figure 5:**
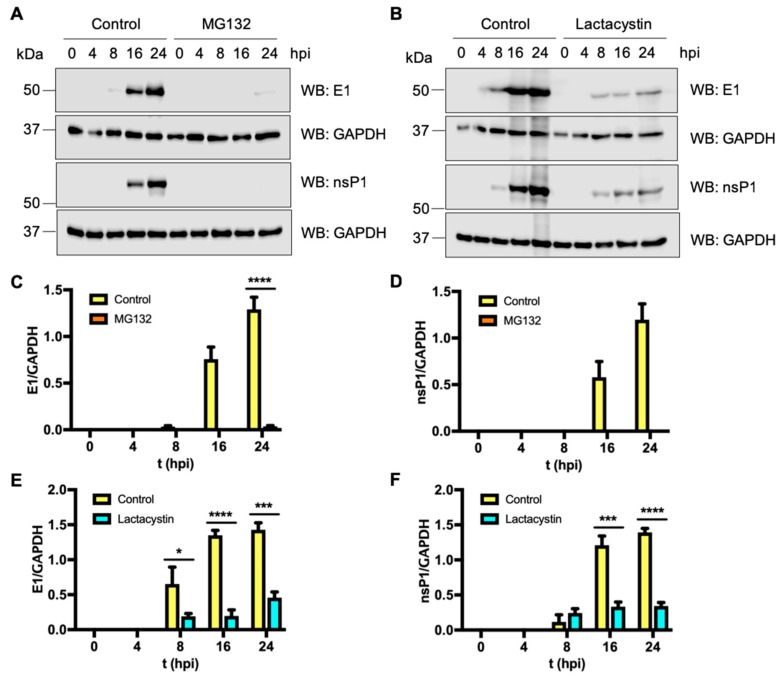
Proteasome inhibition diminished MAYV protein synthesis. Confluent Vero-E6 cells were pre-treated with MG132 (**A**) or Lactacystin (**B**) as described above. In control cells, DMSO (0.1%) or water was added. Cells were then infected with MAYV at an MOI of 1. At different times after infection, the levels of E1 and nsP1 proteins were evaluated by Western blot with anti-E1 and anti-nsP1 antibodies, respectively. Densitometric analysis of Western blot bands was performed using ImageJ software (**C**–**F**). Viral protein expression was normalized to GAPDH. Statistically significant differences are indicated: * *p* < 0.05; *** *p* < 0.001; **** *p* < 0.0001.

To gain insights about the molecular mechanism by which proteasome inhibition affects MAYV replication, we compared viral protein synthesis in infected proteasome inhibitor-treated and untreated cells using Western blot analysis. Since commercial antibodies to detect MAYV proteins are not available, we designed and generated antibodies against the MAYV structural and non-structural proteins E1 and nsP1, respectively (GenScript). To confirm that these antibodies were able to recognize these viral proteins, we performed a Western blot analysis of the recombinant viral proteins using antibodies against E1, nsP1 or pre-immune sera. As shown in Figure S2, the pre-immune sera were unable to detect the recombinant viral proteins (Left panel in A and B). However, the E1 and nsP1 antibodies allowed us to detect bands of the expected size corresponding to recombinants proteins (Right panel in A and B). Then, we decided to test whether the anti-E1 and anti-nsP1 antibodies were able to detect the viral proteins in MAYV-infected cells. Vero cells were infected with the virus at an MOI of 1 and at indicated time points we analyzed the synthetized proteins using Western blot analysis. We did not detect any protein with the pre-immune sera. However, when we used the anti-E1 or anti-nsP1 antibodies, we detected bands with molecular weights corresponding to E1 and nsP1 protein; the bands’ intensity increased with the time of infection (Figure S2, panels C and D). These results indicated that the anti-E1 and anti-nsP1 antibodies could be used to detect both viral proteins by Western blot. We then analyzed the synthesis of E1 and nsP1 proteins at different time points after MAYV infection of proteasome inhibitor-treated or untreated Vero-E6 cells. We detected strong E1 and nsP1 bands at 16 h post infection (hpi) and 24 hpi in untreated cells (Figure 5 panels A and B). MG132 or Lactacystin treatment clearly downmodulated the expression of viral proteins; we found a considerable decrease in the expression of both viral proteins (Figure 5A,B). A densitometric analysis of the bands revealed statistically significant differences in the expression of E1 and nsP1 proteins between treated and untreated cells (Figure 5, panels C, D, E and F).

We performed similar experiments in HeLa cells with similar results (Figure S3). All together these results indicated that proteasome inhibition affects MAYV viral protein synthesis.

### 3.6. Proteasome Activity is also Required for UNAV Replication

**Figure 6:**
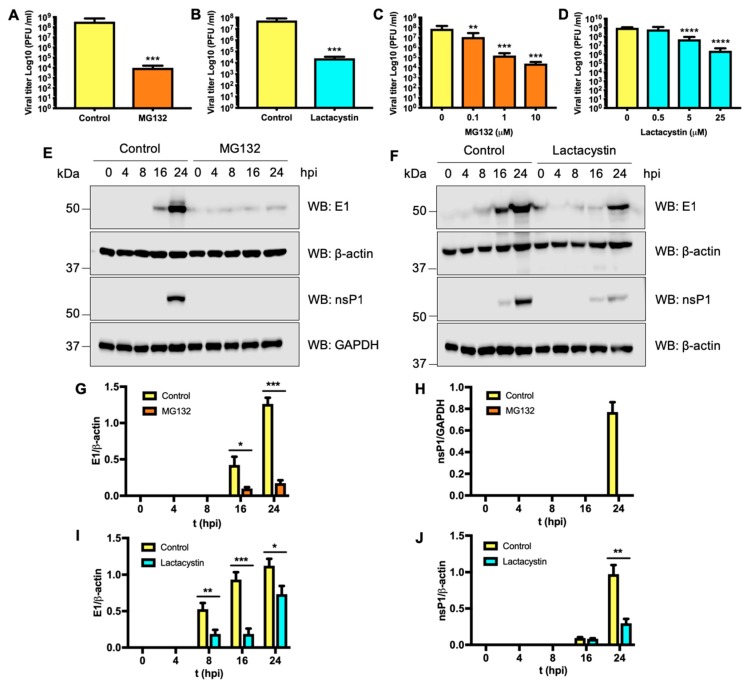
UNAV replication is sensitive to proteasome inhibition. HeLa cells were treated with MG132 (**A**,**C**) or Lactacystin (**B**,**D**), and infected with UNAV at an MOI of 10. In control cells, DMSO (0.1%) or water was added. After 24 h of incubation, viral yields in the cell supernatants were quantified as described above. Densitometric analysis of Western blot bands was performed as described above (**E**–**J**). GAPDH and β-actin antibodies were used to normalize the expression of E1 and nsP1 proteins, respectively. Statistically significant differences are indicated: * *p* < 0.05; ** *p* < 0.01, *** *p* < 0.001.

UNAV is an *Alphavirus* closely related to MAYV that remains poorly characterized [24]. For this reason, we decided to evaluate whether UNAV replication was also sensitive to proteasome inhibition. We first assessed the viral progeny production in HeLa cells infected with UNAV at an MOI of 10 after treatment with MG132 or Lactacystin; untreated cells served as controls. The viral titers observed at 24 h after infection in untreated cells were around 1 × 10^8^ PFU/mL (Figure 6, panels A and B). A significant 3 or 4 log decrease in viral progeny production was found in cells treated with the inhibitors (Figure 6, panels A and B). Moreover, we observed a concentration-dependent downmodulation of the inhibitors (Figure 6, panels C and D). To further explore the consequences of proteasome inhibition on UNAV replication, we decided to evaluate viral protein synthesis as we performed above. UNAV has been grouped within the Selimki Forest antigenic complex, together with other important human pathogenic viruses, such as MAYV, CHIKV, O’nyong-nyong (ONNV), Ross River (RRV), Getah (GETV) and SFV [6]. Since the amino acid sequences of E1 and nsP1 proteins from these viruses show high similarity, we speculated that the polyclonal antibodies generated for MAYV might recognize E1 and nsP1 proteins from other members of this complex. To test this hypothesis, we infected Vero cells with UNAV or CHIKV at an MOI of 1. At different time points after infection, we analyzed the synthesis of E1 or nsP1 proteins. As observed in Figure S4, E1 and nsP1 antibodies were able to recognize both proteins in both UNAV- and CHIKV- infected cells, indicating that these antibodies can be used to study other members of the Selimki Forest complex. Finally, we analyzed the synthesis of E1 and nsP1 in UNAV-infected HeLa cells treated with the inhibitors and compared it with that of untreated cells. As shown in Figure 6 (Panels E to J), we observed a significant reduction in the synthesis of E1 and nsP1 proteins in those cells treated with the proteasome inhibitors. These results indicate that proteasome activity is necessary for UNAV replication.

## 4. Discussion

Viruses employ the host cell machinery and resources to facilitate their replication. Proteasomal degradation pathways play a critical role in regulating multiple protein functions and are essential for many cellular processes including cell cycle progression, immunity and autophagy. Therefore, viruses have evolved to hijack the host proteosomal machinery to improve their replication [32,35,36].

Alphaviruses MAYV and UNAV are emerging pathogens in Latin America, and the essential host factors involved in their replication still remain unknown. In this study, we investigated the role of the UPS in the replication of two neglected viruses. Our results indicate that proteasome inhibition reduces the production of infectious MAYV and UNAV viral particles in a dose-dependent manner. The UPS is involved in the replication of a broad range of viruses such as human respiratory syncytial virus, rotavirus, African swine virus, porcine circovirus type 2 and the *Alphavirus* CHIKV [37,39,40,42,49].

Proteasome inhibitors can affect different steps of the replication cycle, including viral protein expression [50], viral particle maturation [51] and viral budding [52,53]. To determine the stage of viral replication that is sensitive to proteasomal inhibition, we performed a time of addition study. A clear effect was observed when the treatment was given during the immediate early or early phase of infection. Thus, proteasome activity plays a critical role during the initial phase of MAYV infection. In addition, we analyzed whether this inhibition was caused by suppression of viral protein expression. The synthesis of both the structural protein E1 and the non-structural protein nsP1 in MAYV and UNAV was clearly downmodulated in the cells treated with proteasome inhibitors, explaining the reduction in the production of infectious viral particles. Treatment with proteasome inhibitors has been shown to downmodulate the synthesis of rotavirus, vaccinia virus and CHIKV proteins [37,49,54]. Importantly, the FDA-approved proteasome inhibitor Bortezomib, which has been successfully used to treat some types of myeloma and lymphoma, has been shown to be effective in inhibiting other Alphaviruses such as VEEE and EEEV [44,55]. We propose that the UPS is a possible pharmacological target to control MAYV and UNAV infections. It would be interesting to test the efficacy of this drug in an animal model for MAYV or UNAV infection.

## Supplementary Materials

The following are available online at https://www.mdpi.com/1999-4915/11/4/370/s1, Figure S1: Proteasome inhibition promotes the accumulation of K48-polyubiquitinated proteins; Figure S2: Validation of purified antibodies against E1 and nsP1 proteins from MAYV; Figure S3: Proteasome inhibition affects the synthesis of MAYV E1 and nsP1 proteins in HeLa cells; Figure S4: MAYV-purified antibodies recognize E1 and nsP1 from other MAYV-related Alphaviruses such as UNAV and CHIKV.
