# Supplementary material for: A Functional Ubiquitin-Proteasome System is Required for Efficient Replication of New World Mayaro and Una Alphaviruses
Source: Viruses. 2019 Apr 23;11(4):370. doi: 10.3390/v11040370 (PMC6520948; doi:10.3390/v11040370)
Supplement: Supplementary file 1 [file viruses-11-00370-s001.pdf]

viruses

Supplementary material

A functional ubiquitin-proteasome system is required for efficient replication of New World Mayaro and Una Alphaviruses

Yessica Y. Llamas-González [1,2], Dalkiria Campos [1], Juan M. Pascale [3,4], Juan Arbiza [5] and José González-Santamaría [1,4,*]

[1] Grupo de Biología Celular y Molecular de Arbovirus, Instituto Conmemorativo Gorgas de Estudios de la Salud, 0816-02593 Panamá, Panama; qfb.y.llamas@gmail.com (Y.Y.L.-G.); dcampos@gorgas.gob.pa (D.C.)
[2] Programa de Doctorado en Ciencias Biológicas, Universidad de la República, 11200 Montevideo, Uruguay
[3] Dirección de Investigación y Desarrollo Tecnológico, Instituto Conmemorativo Gorgas de Estudios de la Salud, 0816-02593 Panamá, Panama; jmpascale@gorgas.gob.pa (J.M.P.)
[4] Escuela de Medicina, Universidad de Panamá, Panamá, Panama
[5] Sección de Virología, Facultad de Ciencias, Universidad de la República, 11400 Montevideo, Uruguay; jarbiza@fcien.edu.uy (J.A.)
[4] Dirección de Investigación, Universidad Interamericana de Panamá, 9865 Panamá, Panama
* Correspondence: jgonzalezsantamaria@gorgas.gob.pa; Tel.: +507 527-4814

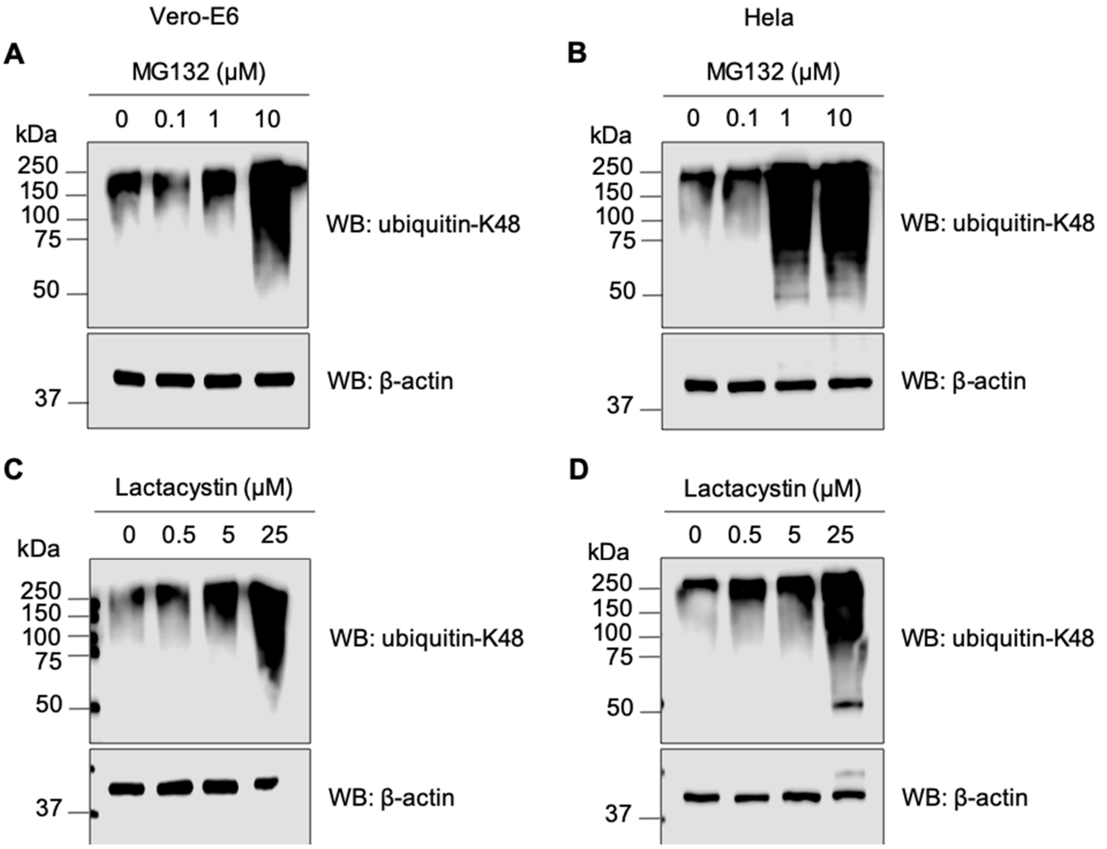

Figure S1. Proteasome inhibition promotes the accumulation of K48-polyubiquitinated proteins. Vero-E6 (**A, C**) or HeLa (**B, D**) cells were treated with MG132 or Lactacystin at the indicated concentrations. In control cells, DMSO (0.1%) or water was added. After 24 h of treatment, the levels of K48-polyubiquitinated proteins were analyzed by Western blot (WB) as described previously. β-actin antibody was used as a loading control.

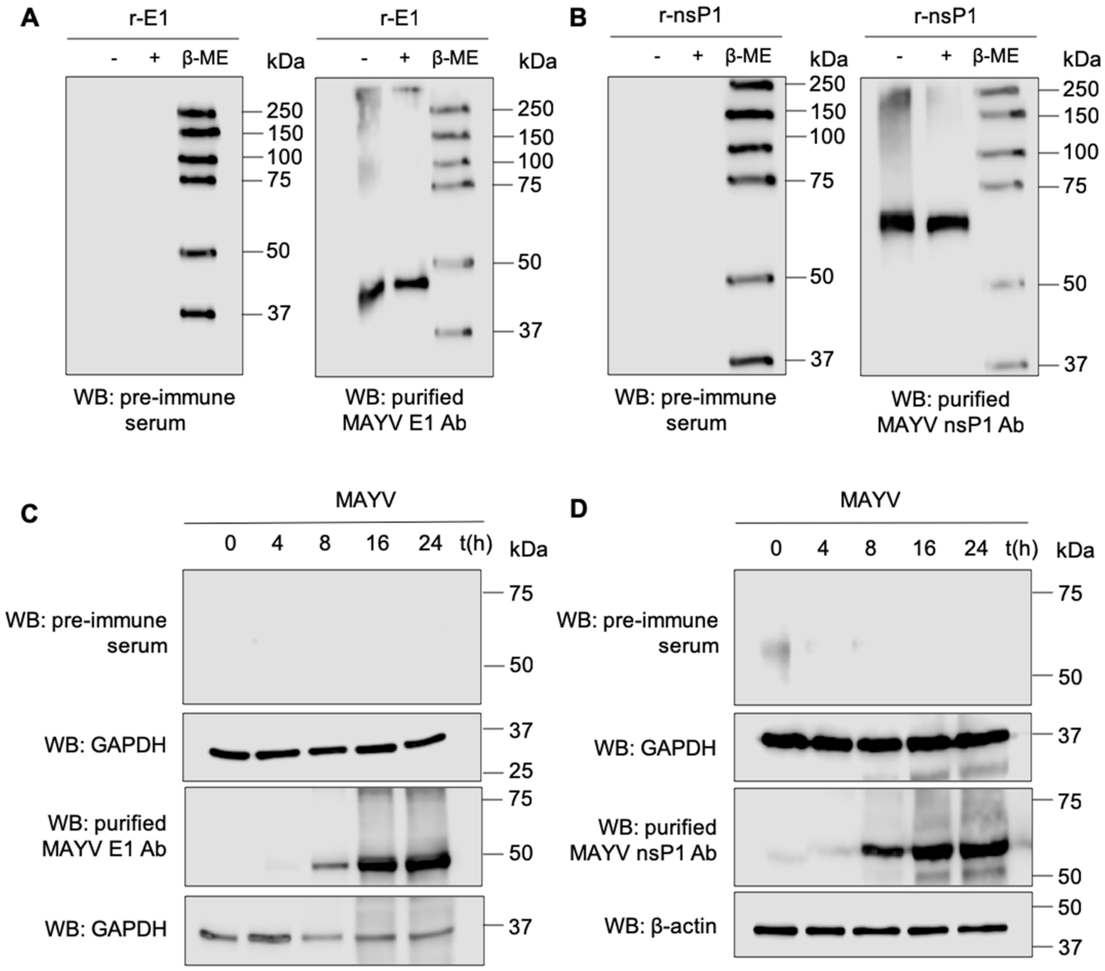

Figure S2. Validation of antibodies against E1 and nsP1 proteins from MAYV. Fifty ng of recombinant MAYV E1 (**A**) or nsP1 (**B**) proteins with or without β-mercaptoethanol (β-ME) were fractionated in SDS-PAGE, transferred to nitrocellulose membranes and incubated with pre-immune sera (left panel in A and B), anti-E1 antibodies or anti-nsP1 antibodies (right panel in A and B) and analyzed by Western blot (WB) as described above. (**C, D**) Vero cells were infected with MAYV at an MOI of 1 and at indicated time points, cells lysates were evaluated with pre-immune sera or antibodies to detect E1 and nsP1 proteins. GAPDH and β-actin antibodies were used as loading controls.

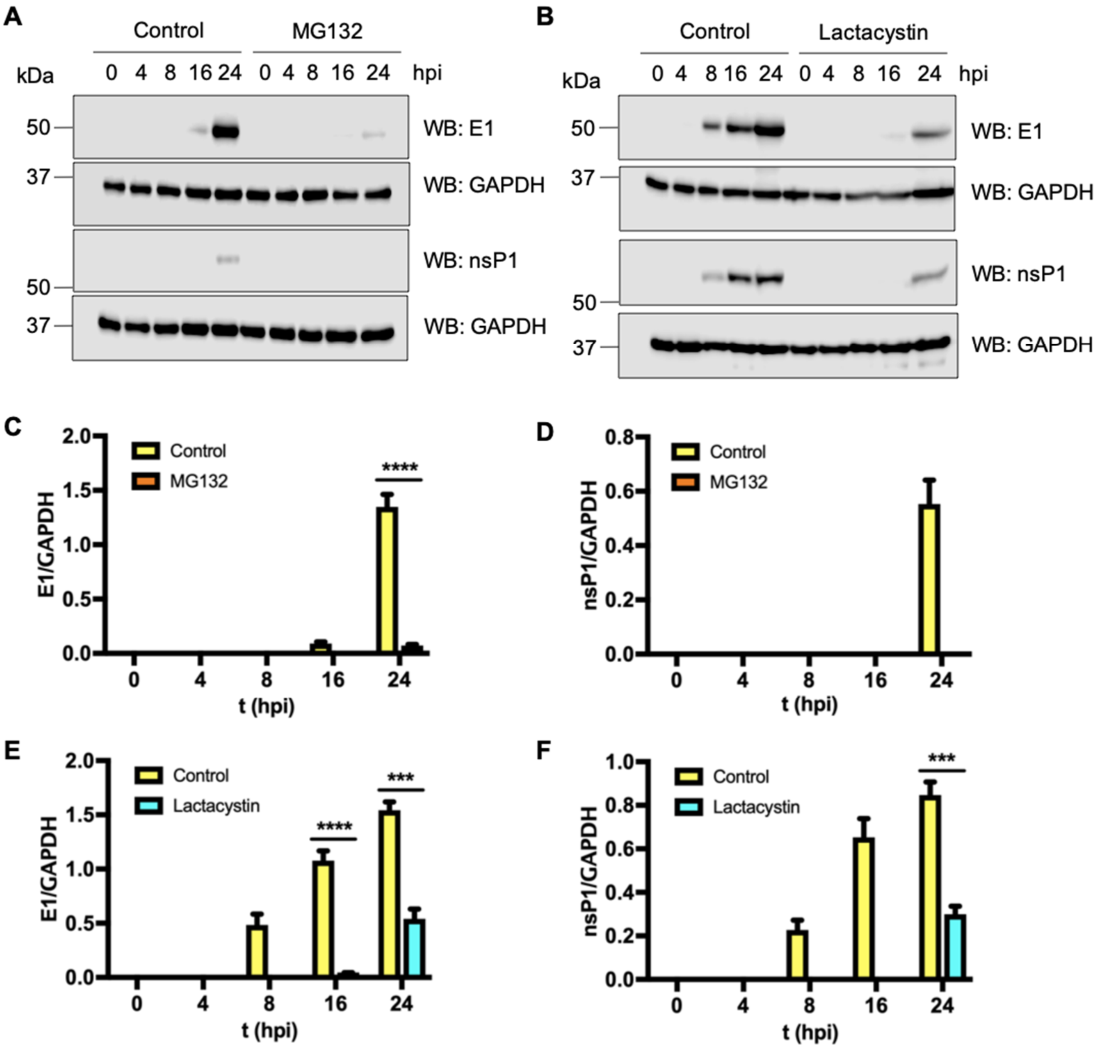

Figure S3. Proteasome inhibition affects the expression of MAYV E1 and nsP1 proteins in HeLa cells. HeLa cells were treated with MG132 (**A**) or Lactacystin (**B**) and infected with MAYV as previously described. Untreated HeLa cells served as controls. In control cells, DMSO (0.1%) or water was added. Cell protein lysates were assessed by Western blot (WB) using anti-E1 and anti-nsP1 antibodies. Quantification of E1 and nsP1 protein expression from cells treated with MG132 (**C**, **D**) or Lactacystin (**E**, **F**) was performed using ImageJ software and normalized against GAPDH as a loading control. Data were analyzed with Two-way ANOVA using GraphPad software and are shown as mean ± SD. Statistically significant differences are indicated: ***$p < 0.001$; ****$p < 0.0001$.

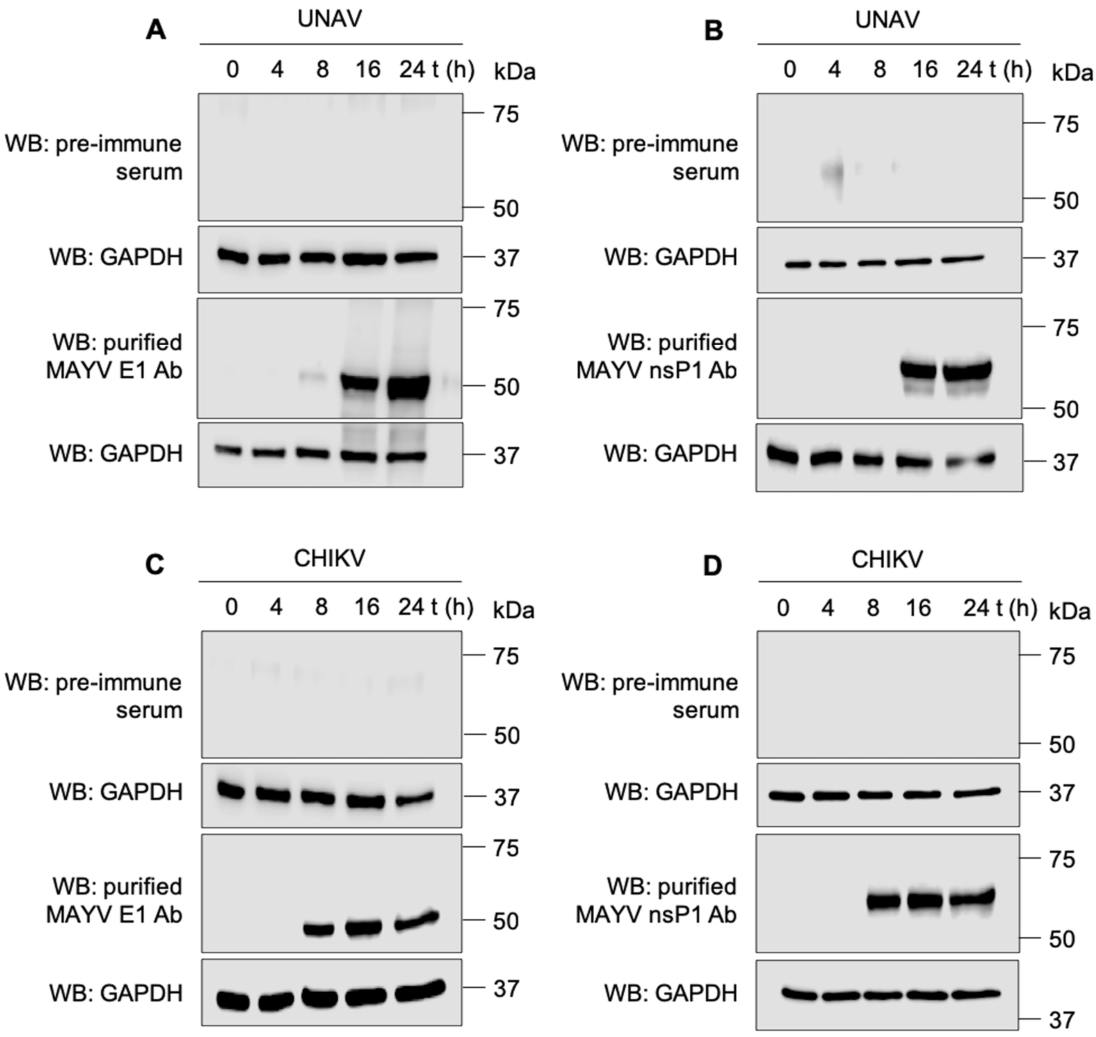

Figure S4. MAYV antibodies recognize E1 and nsP1 from other MAYV-related Alphaviruses, such as UNAV and CHIKV. Vero cells were infected with UNAV (**A**, **B**) or CHIKV (**C**, **D**) at an MOI of 1. At the indicated times, protein cell lysates were evaluated by Western blot (WB) as described previously. GAPDH antibody was used as a loading control.
